# Feruloyl Glyceride Mitigates Tomato Postharvest Rot by Inhibiting *Penicillium expansum* Spore Germination and Enhancing Suberin Accumulation

**DOI:** 10.3390/foods13081147

**Published:** 2024-04-10

**Authors:** Jieyu Gao, Wu Song, Xiaofeng Tang, Yongsheng Liu, Min Miao

**Affiliations:** 1 School of Food and Biological Engineering, Hefei University of Technology, Hefei 230009, China; gaojieyu1995@163.com (J.G.); songwu163@163.com (W.S.); tangxiaofeng1023@hfut.edu.cn (X.T.); 2 School of Horticulture, Anhui Agricultural University, Hefei 230036, China

**Keywords:** natural antimicrobials, feruloyl glyceride (FG), suberin synthesis, postharvest disease control

## Abstract

Postharvest rot, caused by *Penicillium expansum*, in tomatoes poses significant economic and health risks. Traditional control methods, such as the use of fungicides, raise concerns about pathogen resistance, food safety, and environmental impact. In search of sustainable alternatives, plant secondary metabolites, particularly phenolic compounds and their derivatives, have emerged as promising natural antimicrobials. Among these, feruloyl glyceride (FG), a water-soluble derivative of ferulic acid, stands out due to its antioxidant properties, antibacterial properties, and improved solubility. In this study, we provide evidence demonstrating FG is capable of inhibiting the spore germination of *P. expansum* and effectively reducing the incidence rate of *Penicillium* rot of tomatoes, without compromising quality. Electron microscopy observations combined with metabolite and transcriptomic analyses revealed that FG treatments resulted in enhanced suberin accumulation through promoting the expression of suberin synthesis related genes and, consequently, inhibited the growth and expansion of *P. expansum* on the fruits. This work sheds light on the mechanisms underlying FG’s inhibitory effects, allowing its potential application as a natural and safe alternative to replace chemical fungicides for postharvest preservation.

## 1. Introduction

Fungal infection is one of the main reasons leading to postharvest fruit rot, which has considerable economic and health risks; *Penicillium expansum* is one of the fungi infecting tomatoes [1,2]. Traditional fungicide methods of controlling postharvest fruit diseases increase pathogen resistance and threaten food safety and the environment [3,4,5]. In recent decades, the research landscape has shifted towards exploring natural antimicrobials, specifically leveraging plant secondary metabolites as viable alternatives [6]. In plants, a remarkable phenolic-ester-based defense system has evolved and serves as a safeguard against a range of environmental stressors, encompassing drought conditions and pathogenic bacterial infestations [7,8,9]. Notably, suberin, a glycerol-based polymer complex of polylipids and polyphenols, plays a crucial role in this defense mechanism by rapidly accumulating at wound sites to form a protective barrier that significantly reduces water and nutrient loss and hinders further pathogen infiltration [10,11,12,13].

Plant polyphenols, including isovanillic acid, p-hydroxycinnamic acid, syringic acid, caffeic acid, and ferulic acid (FA), have emerged as promising natural bacteriostatic agents, exhibiting broad-spectrum activity against bacteria, fungi, and yeast [14,15,16]. These compounds, renowned for their antioxidant and antimicrobial properties, are widely utilized as natural additives in the food industry [17,18,19]. The application of 1.0 g·L^−1^ ferulic acid has been observed to significantly reduce lesion diameter and colony diameter in *P. expansum*-infected apples in vitro [20]. However, despite their potential, the use of phenolic compounds such as FA still faces practical challenges, including limited water solubility and susceptibility to environmental factors such as light and heat [21,22]. In this context, stable and safe compounds like FA derivatives have gained increasing attention [23,24,25]. Feruloyl glyceride (FG), a water-soluble derivative of FA and an essential monomeric component of suberin, offers a promising alternative [26,27]. FG combines comparable antioxidant and antibacterial properties with enhanced solubility, due to the π-conjugate structure of the phenolic hydroxyl group glycerol skeleton, making it a potential usage as a freshness preservative or antibacterial agent [28,29,30].

In the present study, we aim to investigate the efficacy of FG in inhibiting the growth of *P. expansum* and mitigating the occurrence of *Penicillium* fruit rot in tomatoes, while preserving fruit quality. A comprehensive suite of experiments has been conducted, including plate and fruit inhibition assays, fruit quality detection, transcriptomic sequencing, and metabolite analysis. This research could establish a theoretical foundation for the practical application of FG in extending fruit shelf life, while providing a new perspective to maintain optimal fruit quality.

## 2. Materials and Methods

### 2.1. Microbial Materials and Treatments

The *Penicillium expansum* strain was kindly provided by Professor Samir Droby from the Agricultural Research Organization of the Israeli Ministry of Agriculture [31]. Feruloyl glyceride (FG) was purchased from Sinopharm Chemical Reagent Co., Ltd., Shanghai, China. The strain was inoculated on a Potato Dextrose Agar (PDA) medium with a gradient concentration of FG and was incubated at 25 °C. Colony growth was meticulously observed and documented.

Spore observation: Freshly grown *P. expansum* spores (7–10 d) were harvested and diluted to 10^7^ cfu mL^−1^ with sterile water. Then, 100 μL of spore suspension was added to 20 mL of Potato Dextrose Broth (PDB) medium with a gradient concentration of FG and was incubated at 25 °C on a shaker at 120 rpm. Samples were collected at 0, 4, 8, and 12 h post-incubation and were resuspended in 0.05 M PBS (pH 7.2) buffer. Microscopic analysis assessed spore germination as germ tube length ≥ half of the spore diameter. Each group was replicated thrice, with ≥100 spores observed per replicate. The germination rate was calculated as the ratio of germinated spores to the total count.

### 2.2. Fruits and Treatments

Uniform cherry tomatoes (*Solanum lycopersicum* var. cerasiforme ‘XinTaiyang’) were sourced from the market, free from damage, pests, or diseases. The tomatoes were immersed in the solution of 0.2% (*v*/*v*) sodium hypochlorite for 5 min to surface-disinfect and were then washed with deionized sterile water, before being surface-dried at room temperature. Subsequently, the tomatoes were sprayed evenly with a 20 mM FG solution (approx. 10 mL for 100 fruits). Punched holes about 2 mm in diameter and 3 mm in depth were drilled at the tomato equator with a sterile inoculation spike and tomatoes were inoculated with 1 μL of spore suspension (5 × 10^6^ cfu·mL^−1^). The treated tomatoes were stored in a plastic case at 25 °C and 70% relative humidity. Daily observations and recordings were made.

### 2.3. Measurement of Relative Fungal Biomass

Relative fungal biomass was measured with DNA-based quantitative PCR (qPCR) and the 2^∆CT^ formula [32]. DNA extraction was performed using the cetyltrimethylammonium bromide (CTAB) method, followed by qPCR analysis with the ChamQ SYBR qPCR Master Mix (Vazyme Cat. Q311-02/03, Nanjing, China). The 2^∆CT^ formula was applied to calculate the results, where ∆CT represents the difference in threshold cycle (CT) values between *18SrRNA* (*18Sr*) and the reference gene *SlActin*. Primers are listed in Table S1.

### 2.4. Analysis of Weight Loss and Fruit Firmness

Treated tomatoes were stored at room temperature and observed daily, with fruit weight being measured and recorded throughout. Weight loss during storage was calculated using Equation (1).

(1) $$Weight\;loss(\%)=\frac{W_{0}-W_{t}}{W_{0}}\times 100$$

where W_0_ represents the initial average fruit weight and W_t_ represents the average fruit weight at subsequent time points.

The pericarp puncture resistance of tomato samples was assessed in a texture analyzer (TA-XT PLUS, Stable Micro Systems Ltd., Surrey GU7 1YL, UK). Data were collected from 10 fruits of each group. A P/2N probe was employed to puncture the pericarp twice on the front and opposite sides via the Return to Start method. Testing parameters were listed as follows: pre-test speed: 2.00 mm·s^−1^; test speed: 1.00 mm·s^−1^; post-test speed: 5.00 mm s^−1^; target mode: distance; distance: 10.00 mm; strain: 50.0%; trigger type: auto (force); trigger force: 5.0 g (puncture analysis); probe: P/2N; stop polt at: start position; tare mode: auto; points per second: 200.

### 2.5. Measurement of Soluble Solids Content (SSC) and Titratable Acid (TA)

The soluble solids content of the fruit was measured using a digital refractometer (CNT65, LOHAND Biological, Hangzhou, China). Additionally, the relative content of titratable acid was determined through acid–alkali titration [33].

### 2.6. Determination of Lycopene

Lycopene was extracted using an n-hexane-acetone-ethanol (2:1:1, *v*/*v*) mixture, following the reported method with some slight modifications [34,35]. Briefly, tomato tissues (5.0 g) were homogenized with 50 mL mixture above and 15 mL distilled water with shaking for 10 s, followed by being placed on ice for 10 min in the dark to allow phases to separate. The organic phase was utilized for measuring the absorbance at 503 nm using spectrophotometry, which was then substituted into Equation (2) to calculate lycopene concentration.

(2) $$C_{lycopene}={(A}_{503}\times 31.2)/FW$$

where A_503_ represents the optical density value, FW denotes the fresh weight of the tissues, and the molar extinction coefficient for lycopene is 17.21 mol^−1^ m^−1^.

### 2.7. Determination of Anthocyanin

The total anthocyanin content in the fruit was measured using the pH difference method [36]. Briefly, tomato tissues (0.5 g) after grinding were extracted with 2 mL pH 1.0 buffer (50 mM KCl and 150 mM HCl) and 2 mL pH 4.5 buffer (400 mM sodium acetate and 240 mM HCl), respectively. This was followed by centrifuging at 12,000× *g* for 15 min at 4 °C and the supernatants were analyzed for absorbance at 510 nm. The anthocyanin concentration was calculated using Equation (3).

(3) $$C_{anthocyanin}=\frac{{(A}_{510pH=1.0}-A_{510pH=4.5})\times 484.8}{24,852}$$

where A_510pH=1.0_ and A_510pH=4.5_ represented the absorbance values at pH 1.0 and 4.5, respectively. The constants 484.8 and 24,852 referred to the molecular weight of anthocyanin-3-glucoside chloride and its molar absorption coefficient at 510 nm, respectively.

### 2.8. Microscopy Observation of Suberin Polyphenols

The corkification formation rate was determined by the accumulation of suberin polyphenols (SPPs) on the parenchyma cell wall. Fresh tomato tissues were prepared as frozen slices by smoothing the target tissues with a scalpel, followed by embedding them in an Optimal Cutting Temperature Compound (O.C.T. Compound) on a frozen microtome platform. The embedded samples were sliced into sections with a thickness of 8–10 μm. The SPP spontaneous fluorescence was observed and recorded under a microscope at an excitation wavelength of 488 nm, while the fluorescence intensity was quantified using ImageJ software v1.8.0.345 (W.S. Rasband, National Institutes of Health, Bethesda, MD, USA).

### 2.9. Metabolites Profiling

Metabolite profiling was conducted via a widely targeted metabolite method from Wuhan Metware Biotechnology Co., Ltd., (http://www.metware.cn/, accessed on 21 December 2021), Wuhan, China. Freeze-dried fruit skin samples were powdered and analyzed via UPLC-ESI-MS/MS (UPLC, ExionLC™ AD’ https://sciex.com.cn/, accessed on 21 December 2021; MS, Applied Biosystems 4500 Q TRAP, https://sciex.com.cn/, accessed on 21 December 2021), Shanghai, China, identified by comparing the *m/z* values, the retention time (RT), and the fragmentation patterns with the standards in a self-compiled database [37]. Significantly changed metabolites (SCMs) were filtered according to Variable Importance in Projection (VIP) ≥ 1 and | Log_2_ (Fold Change)|≥ 1 [38]. Data underwent log transformation (log_2_) and mean centering before orthogonal PLS-DA analysis (OPLS-DA). Identified metabolites were annotated with the Kyoto Encyclopedia of Genes and Genomes (KEGG) Compound database and mapped to KEGG Pathways (http://www.kegg.jp/kegg/pathway.html, accessed on 21 December 2021) [39]. Metabolite sets enrichment analysis (MSEA) determined the pathway significance using the *p*-values of hypergeometric tests.

### 2.10. Transcriptomic Profiling

RNA sequencing was performed on the control and FG-treated samples, with RNA purity verification conducted using a NanoPhotometer^®^ spectrophotometer (IMPLEN, Los Angeles, CA, USA). In total, 1 μg RNA per sample was used as input material for the RNA sample preparations. Sequencing libraries were prepared with NEBNext^®^Ultra^TM^ RNA Library Prep Kit for Illumina^®^ (NEB, Ipswich, MA, USA). HISAT software (v2.1.0) mapped the filtered reads to the reference genome, while Feature Counts software (version 1.6.2/StringTie v1.3.4d) was utilized to calculate gene alignment and FPKM. DESeq2 R software (version 1.22.1/edgeR v3.24.3) was used to analyze differentially expressed genes (DEGs) according to False Discovery Rate (FDR) < 0.05 and | log_2_ (Fold Change) | ≥ 1. Gene set enrichment analyses were conducted using GSEA-3.0.jar through the hypergeometric test, while the KEGG and Gene Ontology (GO) enrichment analyses were performed with the Cluster profiler R packages (*p*-value < 0.05) [40]. Protein interaction analysis utilized the STRING database, constructed the network by extracting the target gene list from the database, and visualized the results with IGV (https://igv.org, accessed on 21 December 2021).

### 2.11. Total RNA Isolation and Quantitative Real-Time PCR (qRT-PCR) Analysis

The total RNA of tomato tissue samples was extracted using Trizol reagent (Vazyme Cat. R401-01) based on the manufacturer’s protocol, followed by removing genomic DNAs. The first-strand cDNA was synthesized with oligo(dT)_20_ VN primers using the HiScript III 1st Strand cDNA Synthesis Kit (Vazyme Cat. R312-01/02). qRT-PCR was performed utilizing ChamQ SYBR qPCR Master Mix (Vazyme Cat. Q311-02/03) to determine gene relative expression via the 2^−ΔΔCT^ method [41]. Primers are listed in Table S1 with *SlUBI3* as an internal reference gene.

### 2.12. Statistical Analysis

Statistical significance analyses were conducted using IBM SPSS Statistics 20.0 software. Pairwise comparisons were calculated using Student’s *t*-test (* *p* < 0.05 and ** *p* < 0.01). ANOVA with Duncan’s test was employed for multiple comparisons, where a *p*-value < 0.05 was considered significant.

## 3. Results

### 3.1. FG Inhibits the Growth of P. expansum In Vitro

**Figure 1:**
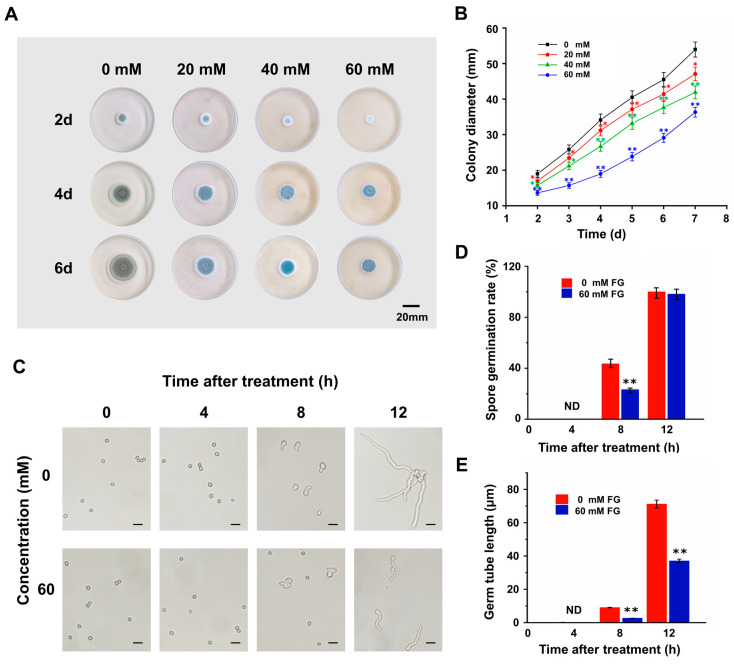
Inhibition of FG on the growth of *P. expansum* in vitro. (**A**) Growth state of *P. expansum* on PDA medium with different concentrations of FG. Bar = 20 mm. (**B**) Statistics of colony diameter. (**C**) Microscopic observation of *P. expansum* spore germination status. Bar = 20 μm. (**D**) Statistics of spore germination rate. (**E**) Statistics of germ tube length. Values in (**B**,**D**,**E**) are means ± SD (n = 6), analyzed using Student’s *t*-test, * *p* < 0.05, ** *p* < 0.01. ND means no data.

The effect of feruloyl glyceride (FG) on the growth of *P. expansum* in vitro was observed on the PDA plate. Experimental data displayed that the addition of FG to the PDA medium resulted in a substantial suppression of *P. expansum* growth in a dose-dependent manner (Figure 1A,B). Furthermore, the effect of FG on the spore germination and germ tube elongation of *P. expansum* was examined via microscopic observation and the results showed that FG significantly suppressed spore germination and germ tube elongation of *P. expansum* (Figure 1C). About 48% of the spores of *P. expansum* germinated after 8 h under normal incubation. In contrast, only 23% of the spores germinated after applying FG, resulting in a 2-fold reduction in the spore germination rate (Figure 1D). Moreover, FG remarkably inhibited germ tube elongation, with approximately one-third of that being in the control group at 8 h and merely half of that at 12 h (Figure 1E).

These results indicated that FG was capable of suppressing *P. expansum* in vitro via inhibiting spore germination and germ tube elongation, providing a viable basis for the subsequent study of FG application in controlling postharvest *Penicillium* fruit rot in tomatoes.

### 3.2. FG Suppresses Penicillium Rot of Tomato Fruits without Affecting the Quality

**Figure 2:**
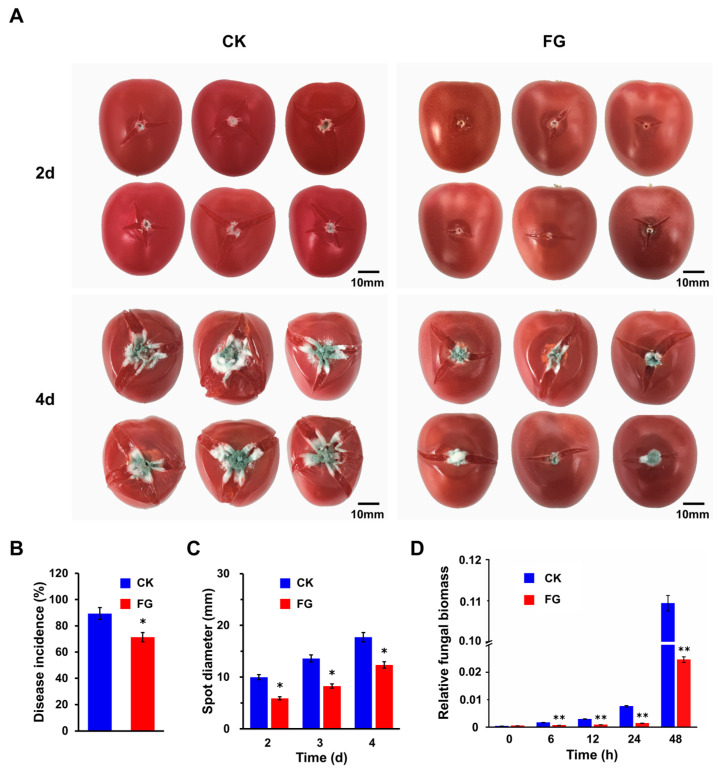
Inhibition of FG against *Penicillium* rot of tomato fruit. (**A**) Cherry tomato fruits after inoculation with *P. expansum* spores. Bar = 10 mm. (**B**) Statistics of disease incidence. (**C**) Statistics of spot diameter on tomatoes. (**D**) Relative fungal biomass. The growth of *P. expansum* was quantified using qPCR [2^[CT (*SlActin*) − CT (*18Sr*)]^]. FG: the FG-treated group; CK: the control check group. Values in (**B**,**C**) are means ± SD (n = 100), values in (**D**) are means ± SD (n = 3), analyzed using Student’s *t*-test, * *p* < 0.05, ** *p* < 0.01.

After determining that FG could inhibit *P. expansum* in vitro, we focused on its application in preventing *Penicillium* fruit rot in tomatoes. The results showed that the cherry tomato exhibited disease symptoms two days after spore inoculation. By the fourth day, the lesions had progressed to encompass nearly the entire fruit, ultimately causing its collapse (Figure 2A). Notably, the severity of the disease and the degree of fruit collapse were lower in the FG-treated (FG) group compared to the control check (CK) group, highlighting the effectiveness of FG in reducing the incidence of tomato rot. Specifically, the CK group exhibited an incidence rate of approximately 89.40% two days post-inoculation, whereas the FG group’s incidence rate was approximately 71.43% at the same time point, resulting in a reduction of about 17.97% (Figure 2B). Analysis of lesion diameters on diseased fruits further corroborated these findings, revealing that the average lesion size in the CK group was approximately 9.99 mm, 13.60 mm, and 17.72 mm at 2, 3, and 4 d post-inoculation, respectively. In contrast, the FG group exhibited average lesion sizes of approximately 5.90 mm, 8.27 mm, and 12.34 mm, respectively, which was significantly reduced (Figure 2C).

Additionally, as the early stage biomass of *P. expansum* on tomato fruits cannot be accurately determined solely based on lesion size, due to the lag time of at least 30 h from spore inoculation to visible lesion formation, a DNA-based qPCR analysis was employed to quantify the relative biomass of *P. expansum* on the fruits. The results demonstrated that no significant changes were observed in the relative biomass of *P. expansum* from 0 to 6 h post-inoculation, followed by a gradual increase from 6 to 24 h and a subsequent substantial elevation. Notably, the relative biomass of *P. expansum* in the FG group was significantly lower compared to the control (Figure 2D), which is consistent with the findings from the in vitro inhibition experiments. The application of FG as an antimicrobial agent against *Penicillium* fruit rot raises the question of its potential impact on fruit quality. Therefore, a comprehensive series of tests were conducted to assess vital quality indicators, including weight loss, hardness, compactness, soluble solids content (SSC), titratable acidity (TA), lycopene, and anthocyanin content. According to the findings, no statistically significant disparities were observed in the fruit quality between the FG group and the CK group, indicating that both groups exhibited similar levels of fruit quality during the storage period (Figure S1).

### 3.3. FG Enhances Suberin Accumulation on the Wound Surface

**Figure 3:**
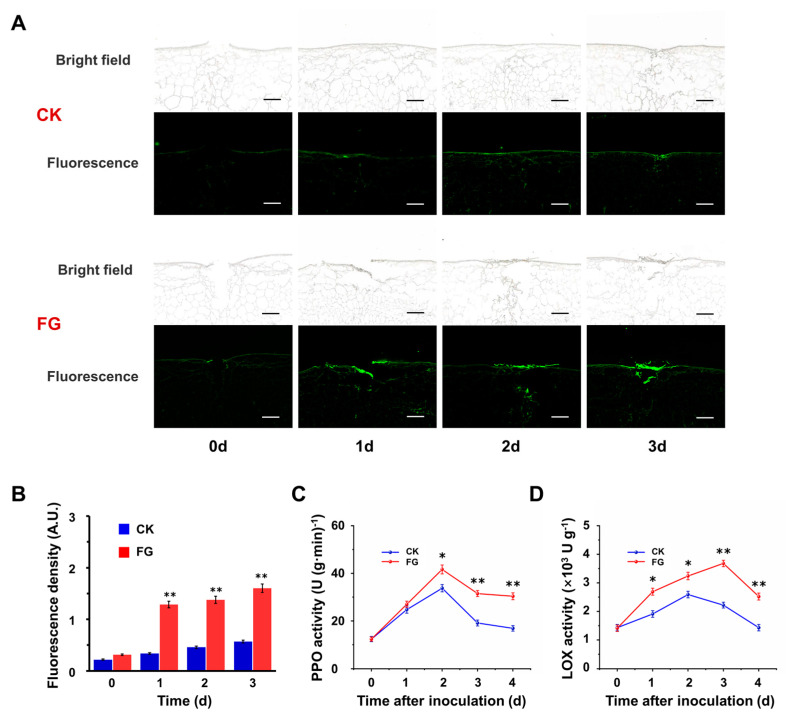
Promotion of FG on the accumulation of suberin. (**A**) Autofluorescence of suberin polyphenolics (SPPs) at the inoculation site of tomatoes. Spontaneous fluorescence of SPPs at 488 nm excitation wavelength, bar = 500 μm. (**B**) ImageJ quantitative mean fluorescence density. (**C**) Polyphenol oxidase (PPO) activity. (**D**) Lipoxygenase (LOX) activity. FG: the FG–treated group; CK: the control check group. Values in (**B**–**D**) are means ± SD (n = 3), analyzed using Student’s *t*–test, * *p* < 0.05, ** *p* < 0.01.

Given that FG is a crucial monomeric constituent of suberin, a thorough examination was conducted to elucidate the effects of FG treatment on suberin deposition. Suberin polyphenols (SPPs), being the primary components of suberin and exhibiting autofluorescence at 488 nm under microscopic examination, were utilized as indicators for evaluating suberin accumulation. Microscopic analyses conducted on the obtained slices within the initial 0–3 d post-wounding demonstrated the progressive accumulation of SPPs on the wound surface, with a notable increase in the fluorescence intensity observed in the FG group compared to the control (Figure 3A). Quantitative statistics show that in the first three days after inoculation, the fluorescence density of the FG group was 3.84 times, 3.02 times, and 2.82 times higher than that of the CK group, respectively (Figure 3B). Moreover, enzyme activity assays conducted on tissues surrounding the inoculation site showed elevated levels of polyphenol oxidase (PPO) and lipoxygenase (LOX) activities post-inoculation, with the FG group consistently demonstrating significantly higher enzyme activities compared to the control (Figure 3C,D). These results suggest that FG stimulates suberization, facilitating accelerated wound healing.

### 3.4. Impact of FG on Differential Modulation in Suberin Metabolites

**Figure 4:**
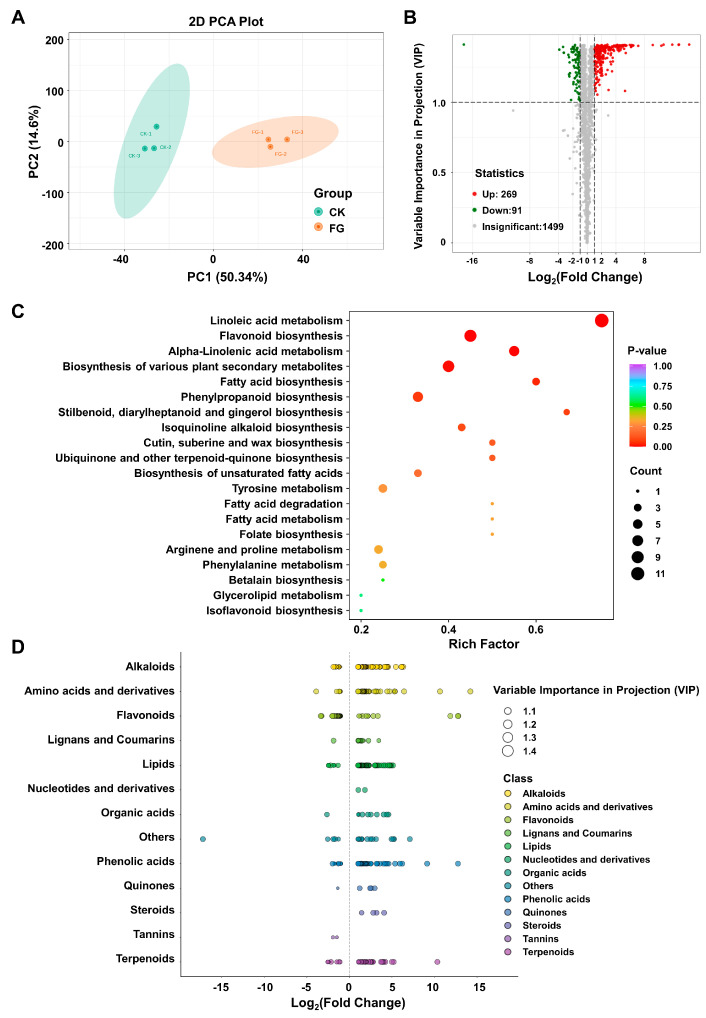
Metabolic profile change of tomatoes in response to FG via a widely targeted metabolomic sequencing. (**A**) Grouping principal component analysis diagram. PC1 represents the first principal component, PC2 represents the second principal component, and percentage represents the interpretation rate of the principal component to the data set; each point in the graph represents a sample, as follows: green represents the CK group and orange represents the FG group. (**B**) Volcano plot of differential metabolites. Each point represents a metabolite, with green, red, and gray indicating downregulated metabolites, upregulated metabolites, and metabolites with insignificant differences, respectively. The x–axis represents the log_2_ fold change (log_2_FC) of the relative abundance of a metabolite between two sample groups. A larger absolute value on the x-axis indicates a greater difference in the relative abundance of the metabolite between the two groups. The y-axis represents the variable importance in projection (VIP), with higher values indicating more significant differences and a more reliable identification of differential metabolites. (**C**) KEGG enrichment plot of differential metabolites. The x–axis represents the rich factor corresponding to each pathway, while the y-axis shows the pathway names sorted by *p*-value. The color of the dots reflects the *p*-value, with redder colors indicating a more significant enrichment. The size of the dots represents the number of enriched differential metabolites. (**D**) Scatter plot of differential metabolites. Each point in the figure represents a metabolite, with different colors indicating different classifications. The x-axis represents the log_2_FC of the relative abundance difference of a substance between the two sample groups. The larger the absolute value of the x-axis, the greater the difference in the abundance of the substance between the two sample groups. The size of the dot represents the VIP value.

To substantiate the beneficial effects of FG on suberin accumulation, an in-depth analysis of metabolite patterns induced by FG was conducted. Metabolites were extracted from fruits inoculated with *P. expansum* spores from the CK and FG groups after 30 h and were, subsequently, analyzed using UPLC–MS/MS. The metabolite profiles of samples were then subjected to principal component analysis (PCA). The score plots of PCA exhibited two principal components (PC1 and PC2), accounting for 50.34% and 14.64%, respectively. The PC1 value indicated discriminative metabolic levels between the FG sample and the CK groups (Figure 4A). By setting the threshold for significantly differential metabolites (DMs) screening at variable importance in projection (VIP) > 1.0 and a *p*-value < 0.05, a total of 360 DMs were obtained, of which 269 DMs were significantly upregulated and 91 DMs were significantly downregulated, respectively (Figure 4B).

The Kyoto Encyclopedia of Genes and Genomes (KEGG) pathways were organized and analyzed for insights into the functional roles of FG on the identified DMs in tomato metabolism. (Figure 4C). The point’s color corresponds to the *p*-value of the hypergeometric test, where lower values indicate more reliable and statistically significant results. Additionally, the dot size indicates the number of differential metabolites in the corresponding pathway, with a larger size reflecting a higher significance of these differences. It could be seen that the DMs were mainly enriched in the following six pathways: “Linoleic acid metabolism”, “Flavonoid biosynthesis”, “alpha-Linolenic acid metabolism”, “Biosynthesis of various plant secondary metabolites”, “Fatty acid biosynthesis”, and “Phenylpropanoid biosynthesis” (Figure 4C). Thirteen categories were identified based on the molecular structure and function of the DMs (Figure 4D). The top five categories were alkaloids (61), lipids (58), flavonoids (52), amino acids and derivatives (50), and phenolic acids (49) (Figure 4D, Table S2). Among them, several compounds involved in the synthesis pathway of suberin substances were notably upregulated in the FG group, including coniferyl alcohol, phenylalanine, coumaric acid, ferulic acid, methyl ferulate, and oleic acid (Table S3). Collectively, the metabolome analysis outcomes offer robust evidence indicating that FG treatment markedly augments the biosynthesis of secondary metabolites associated with suberization.

### 3.5. FG Stimulates the Transcription and Expression of Suberin-Related Genes

**Figure 5:**
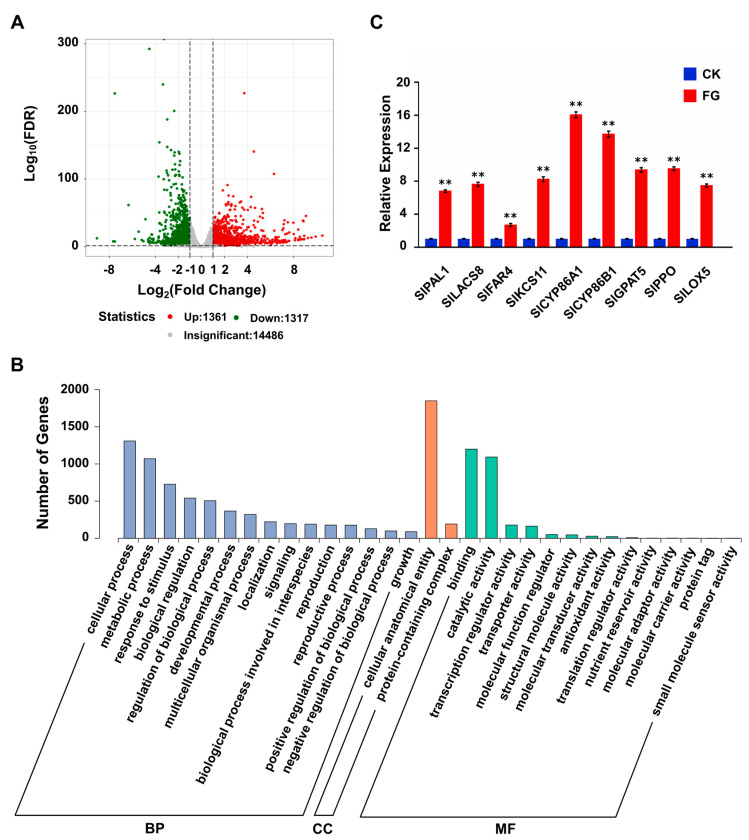
The effect of FG on the expression of genes related to suberization. (**A**) Volcano plot of differential genes. The x-axis represents the fold change in gene expression, while the y-axis indicates the significance level of differential genes. Red, green, and gray dots represent upregulated, downregulated, and non-differentially expressed genes, respectively. (**B**) qRT-PCR analysis of critical genes involved in regulating the biosynthesis of suberin. *SlUBI3* (Solyc01g056940) was regarded as the internal control. FG: the FG-treated group; CK: the control check group. Values are means ± SD (n = 3), analyzed using Student’s *t*-test, ** *p* < 0.01. (**C**) GO functional enrichment analysis. GO is divided into three parts, as follows: biological processes (BPs), cellular components (CCs), and molecular functions (MFs). The x-axis represents the secondary GO terms, while the y-axis indicates the number of differential genes in each GO term.

To investigate the impact of FG on the tomato transcriptome and its correlation with the observed metabolic alterations, RNA-sequencing (RNA-Seq) analysis was carried out on the same samples used for metabolic analysis (the raw sequence data were deposited in the SRA database of NCBI with BioProject SUB14285129). The correlation index r (Pearson’s Correlation Coefficient) for three biological replicates from each sample was close to 1, indicating reliable RNA-seq data (Figure S2). The analysis of differentially expressed genes (DEGs), based on a cut-off threshold of | log_2_ (Fold Change) | ≥ 1 and *p*-value < 0.05, demonstrated that the FG group exhibited 1361 upregulated genes and 1317 downregulated genes in comparison to the CK group (Figure 5A).

Gene ontology (GO) analysis indicated that FG treatment influenced multiple metabolic pathways, including cellular process, metabolic process, response to stimulus, biological regulation, regulation of the biological process, cellular anatomical entity, binding, and catalytic activity (Figure 5B). Delving deeper into the DEGs uncovered that a series of key genes involved in the suberin biosynthesis pathway were upregulated in response to FG treatment (Table S4). The qRT-PCR analysis further corroborated these findings, demonstrating that the transcript levels of key genes (*SlPAL1*; *SlLACS8*; *SlFAR4*; *SlKCS11*; *SlCYP86A1*; *SlCYP86B1*; *SlGPAT5*; *SlPPO*; and *SlLOX5*) were significantly elevated in the FG group compared to the CK group, suggesting that the RNA-Seq data are reliable and that FG stimulates suberization by regulating the transcription of suberin biosynthesis-associated genes (Figure 5C).

**Figure 6:**
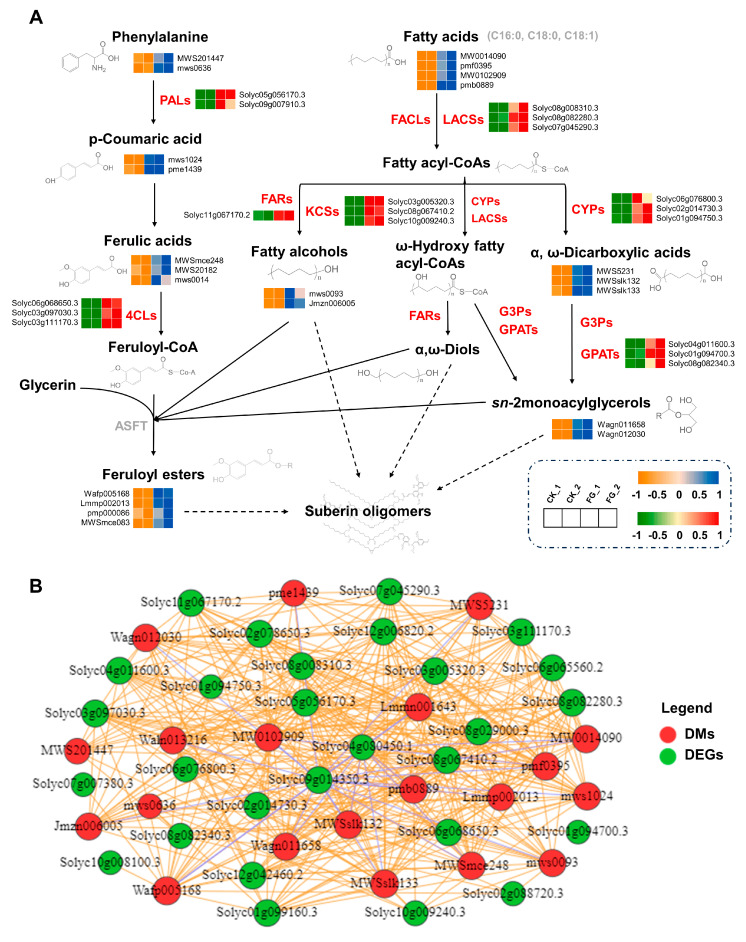
Analysis of suberin biosynthesis pathway and metabolic network. (**A**) Schematic of suberin biosynthesis pathway. Differentially expressed genes and metabolites are marked next to related enzymes or products in the form of a heat map. The color scale represents the values of the log_2_ ratio (FG/CK), with metabolites ranging from orange (low) to blue (high) and genes ranging from green (low) to red (high). (**B**) The connection network of suberin metabolism-related genes and metabolites. Red circles represent differential metabolites (DMs), while green circles represent differentially expressed genes (DEGs). Yellow lines indicate positive correlations and blue lines indicate negative correlations.

A combined metabolic and transcriptomic analysis was helpful to visualize the effects of FG on the suberin synthesis pathway (Figure 6A). To be specific, critical genes in this pathway, such as *PAL* (Solyc05g056170.3, Solyc09g007910.3); *GPAT* (Solyc04g011600.3, Solyc01g094700.3, Solyc08g082340.3); *LACS* (Solyc08g008310.3, Solyc08g082280.3, Solyc07g045290.3); *KCS* (Solyc03g005320.3, Solyc08g067410.2, Solyc10g009240.3); *4CL* (Solyc06g068650.3, Solyc03g097030.3, Solyc03g111170.3); *FAR* (Solyc11g067170.2); and *CYP86* (Solyc06g076800.3, Solyc02g014730.3, Solyc01g094750.3), exhibited significant upregulation in response to FG treatment (Figure 6A, Table S5). Concordantly, the trends in the suberin biosynthesis pathway indicated an augmentation in both metabolic intermediates and end products, such as fatty alcohols (mws0093, Jmzn006005), phenylalanine (MWS201447, mws0636), p-coumaric acid (mws1024, pme1439), ferulic acids (MWSmce248, MWS20182, mws0014), feruloyl esters (Wafp005168, Lmmp002013, pmp000086, MWSmce083), fatty acids (MW0014090, pmf0395, MW0102909, pmb0889), sn-2-monoacylglycerols (Wagn011658, Wagn012030), and α, ω-dicarboxylic acids (MWS5231, MWSslk132, MWSslk133) (Figure 6A, Table S6). A Pearson correlation analysis was conducted between 38 DEGs and 22 DMs related to suberin metabolism to further delineate the relationship between genes and metabolites. (Figure 6B). Notably, 28 DEGs exhibited strong positive and negative correlation coefficient values (R^2^ > 0.8 or <−0.8 and a *p*-value < 0.05) with 20 DMs, with a strong positive correlation between most of the key genes and metabolites involved in suberin synthesis. (Figure 6B, Table S7). Our findings indicate that FG stimulates the transcription and expression of genes related to suberin synthesis, thereby promoting the generation and accumulation of suberin. This, in turn, suggests a potential mechanism by which FG suppresses the *Penicillium* rot of tomato fruits by modulating the suberin synthesis pathway.

## 4. Discussion

During the storage process postharvest, decay caused by fungal infections is a serious problem in tomato crops [3]. In our study, investigating natural antimicrobials to combat postharvest tomato decay caused by *Penicillium expansum*, feruloyl glyceride (FG) was identified as a water-soluble phenolic acid glyceride with antioxidant and antibacterial properties. Our experiments demonstrated that FG is capable of inhibiting spore germination of *P. expansum* and effectively reducing the incidence of *Penicillium* rot in tomatoes, without affecting their quality. Further analysis has revealed that FG can promote the expression of genes related to suberin synthesis, thereby promoting the accumulation of suberin and inhibiting the growth and further invasion of *P. expansum* on the fruit.

In vitro studies demonstrate that FG exhibits a concentration-dependent inhibitory effect on the growth of *P. expansum*. Detailed analysis of colony morphology reveals that the primary inhibitory action of FG occurs during the initial phases of colony development, manifesting as a significant reduction in colony diameter and attenuation of colony pigmentation (Figure 1A). These phenotypic changes are primarily attributed to the suppression of spore germination and subsequent reduction in mycelial growth. From a structural perspective, the phenolic hydroxyl group of FG confers antioxidant capacity, whereas the glyceryl ester group enhances its solubility (Figure S3). Analogous phenolic acid compounds, such as ferulic acid (FA) and its derivatives, are known to interact with the cellular membranes of pathogens, disrupting their structural integrity and, thereby, impeding spore germination [42,43]. This interaction is hypothesized to occur via two primary mechanisms. One proposed mechanism is that the hydroxyl (-OH) groups serve as the hydrophilic moieties to achieve sufficient solubility within the lipid bilayer of the cell membrane, inducing conformational changes in the membrane that potentially alter its permeability and function [44]. The other mechanism is that dissociating phenolic acids like FG within the membrane environment may lead to hyperacidification, which modifies the cell membrane potential. This altered potential disrupts the function of the sodium–potassium pump, a critical component of cellular homeostasis [45]. Specifically, in conjunction with a system of π-delocalized electrons from the benzyl moiety, the phenolic -OH group facilitates the easy loss of protons from the -OH group, leading to a decrease in pH and subsequent protein denaturation. The cumulative effect of these mechanisms ultimately compromises cell membrane integrity, resulting in ion leakage and cell death [46].

Fruit antifungal experiments have demonstrated that FG can effectively reduce fruit incidence, an effect that may be attributed to its ability to promote suberin accumulation. Based on our experimental and multi-omics analysis results, the suberization-promoting effect of FG may be associated with an enhanced responsiveness to stimulus, modulation of plant hormones, and augmented plant immune signaling responses. Previous studies reported that FA can stimulate the production of reactive oxygen species (ROS), which are the primary molecules generated during stress in organisms and can also induce suberization [20,47]. Furthermore, ROS can stimulate the production of plant defense hormones such as abscisic acid (ABA), jasmonic acid (JA), and salicylic acid (SA), which, in turn, can also contribute to further ROS accumulation [48,49]. ABA, known as a stress hormone, is frequently induced under adverse environmental conditions. ABA has been shown to stimulate suberization through the activation of PAL, cinnamyl alcohol dehydrogenase (CAD), and peroxidase (POD) to accelerate wound-healing of wounded kiwifruit [50]. JA and methyl jasmonate (MeJA), as plant hormones and signal molecules associated with damage, can stimulate the expression of resistance genes [51]. Research has demonstrated that exogenous MeJA treatment can enhance tomato resistance to *Botrytis cinerea* [52]. Additionally, MeJA can activate the expression of genes involved in phenolic acid biosynthesis (*PAL1*, *C4H1*, *4CL1*, and *CYP98A14*), promoting the synthesis and accumulation of phenolic acids [53]. When pathogens infect plants, SA is synthesized quickly and exerts antibacterial effects by triggering the expression of pathogenesis-related (PR) proteins, as well as antioxidant enzymes. SA is usually stored in vacuoles as salicylic acid-2-O-β-glucoside (SAG) [54]. Transcriptome analysis revealed that the transcription level of the respiratory burst homolog *RbohB*, a key gene for ROS production, was upregulated in the FG group compared to the control (Table S4). Additionally, metabolomics data analysis showed increased levels of ABA, JA, MeJA, and SAG in the FG group, with the most significant increase in MeJA (Figure S4). Based on these findings, we hypothesize that FG may amplify the signals of the defense response by stimulating the production of ROS and defense-related hormones, particularly JA, thereby promoting the synthesis of suberin and enhancing fruit resistance.

Although our experiments have demonstrated the inhibitory effect of FG on Penicillium fruit rot, complete eradication remains elusive (Figure 2), which may be related to the concentration and freshness of penicillium spores used in the experiments. Therefore, there is still a long way to go in the practical application of FG in antimicrobial preservation for tomatoes. This involves designing experiments to observe the impact of FG on the natural decay of postharvest tomatoes; exploring FG’s resistance to other fungi that tomatoes are prone to, such as *Botrytis cinerea*; investigating the antimicrobial spectrum of FG; and combining FG with existing antifungal agents to seek the appropriate proportions for the development of safer and more efficient antimicrobial preservation compounds.

## 5. Conclusions

In this study, we have investigated the potential of feruloyl glyceride (FG) in controlling postharvest *Penicillium* rot in tomatoes. Our results demonstrate that FG effectively inhibits spore germination of *Penicillium expansum*, significantly reducing the incidence of rot, without deteriorating the fruit quality. Furthermore, our findings imply that FG promotes the deposition of suberin, which contributes to its antimicrobial activity. This research offers valuable insights into the development of sustainable and safe alternatives to replace traditional fungicides for postharvest disease control in tomatoes.

## Data Availability

The original contributions presented in the study are included in the article/supplementary material, further inquiries can be directed to the corresponding authors.

## Supplementary Materials

The following supporting information can be downloaded at: https://www.mdpi.com/article/10.3390/foods13081147/s1, Figure S1. FG had no significant effect on postharvest storage and fruit quality; Figure S2. Sample correlation graph of transcriptome sequencing; Figure S3. Chemical structural formula of FG; Figure S4. Effects of FG on defense hormones; Table S1: The sequence of qPCR primers; Table S2. Quantities of various compounds; Table S3. Differential metabolites with upregulated expression; Table S4. Differentially expressed genes with upregulated expression; Table S5. Differentially expressed genes in the suberin synthesis pathway; Table S6. Differential metabolites in the suberin synthesis pathway; Table S7. Correlation analysis.
