# Supplementary material for: Feruloyl Glyceride Mitigates Tomato Postharvest Rot by Inhibiting Penicillium expansum Spore Germination and Enhancing Suberin Accumulation
Source: Foods. 2024 Apr 10;13(8):1147. doi: 10.3390/foods13081147 (PMC11049243; doi:10.3390/foods13081147)
Supplement: Supplementary file 1 [file foods-13-01147-s001.zip › Supplemental Figures.pdf]

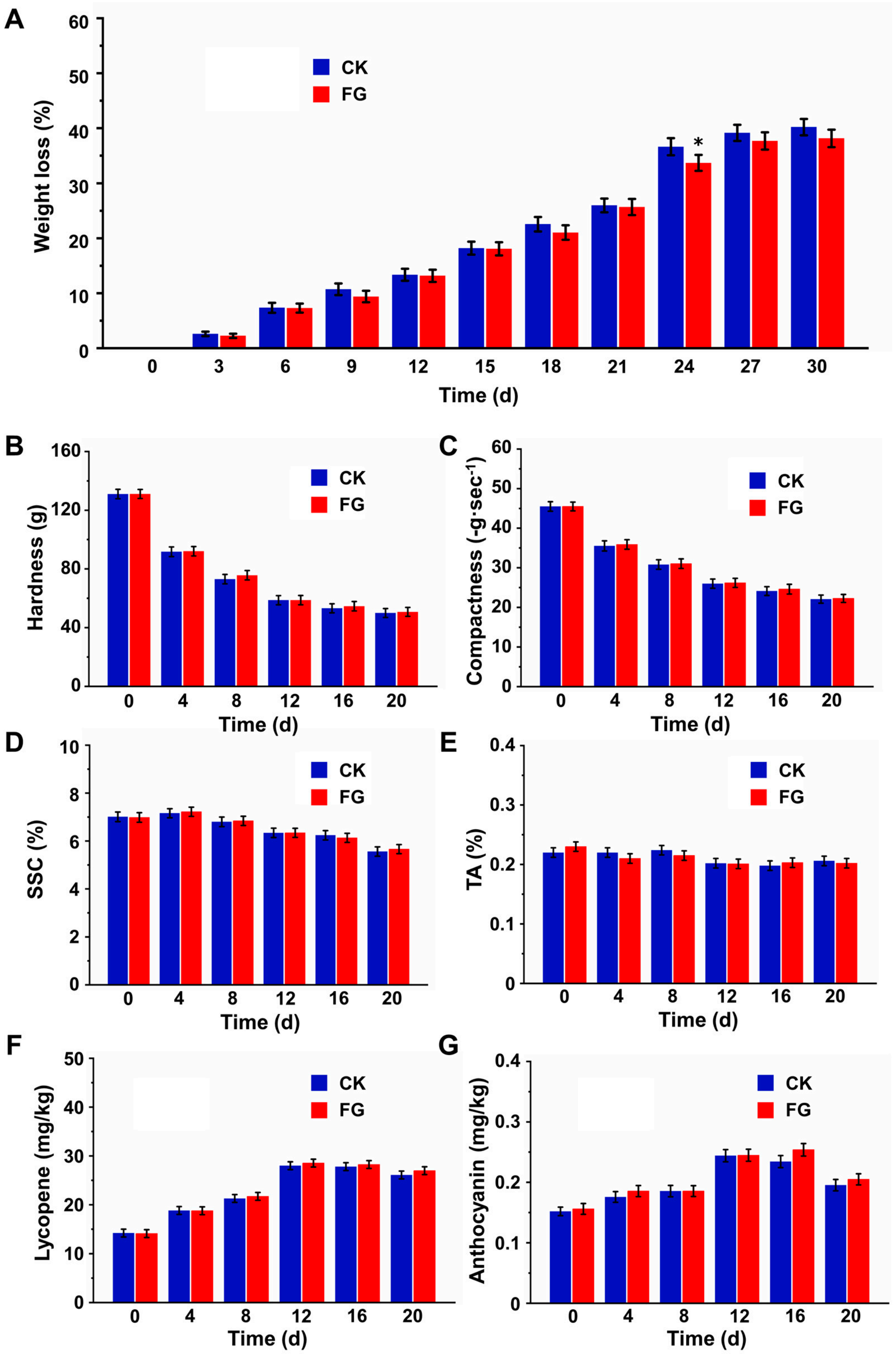

Figure S1. FG had no significant effect on postharvest storage and fruit quality. (A) Weight loss. (B) Hardness. (C) Compactness. (D) Soluble solids content (SSC). (E) Titratable acidity (TA). (F) Lycopene content. (G) Anthocyanin content. Values are means ± SD (n = 10), analyzed by Student's t-test, * $p < 0.05$.

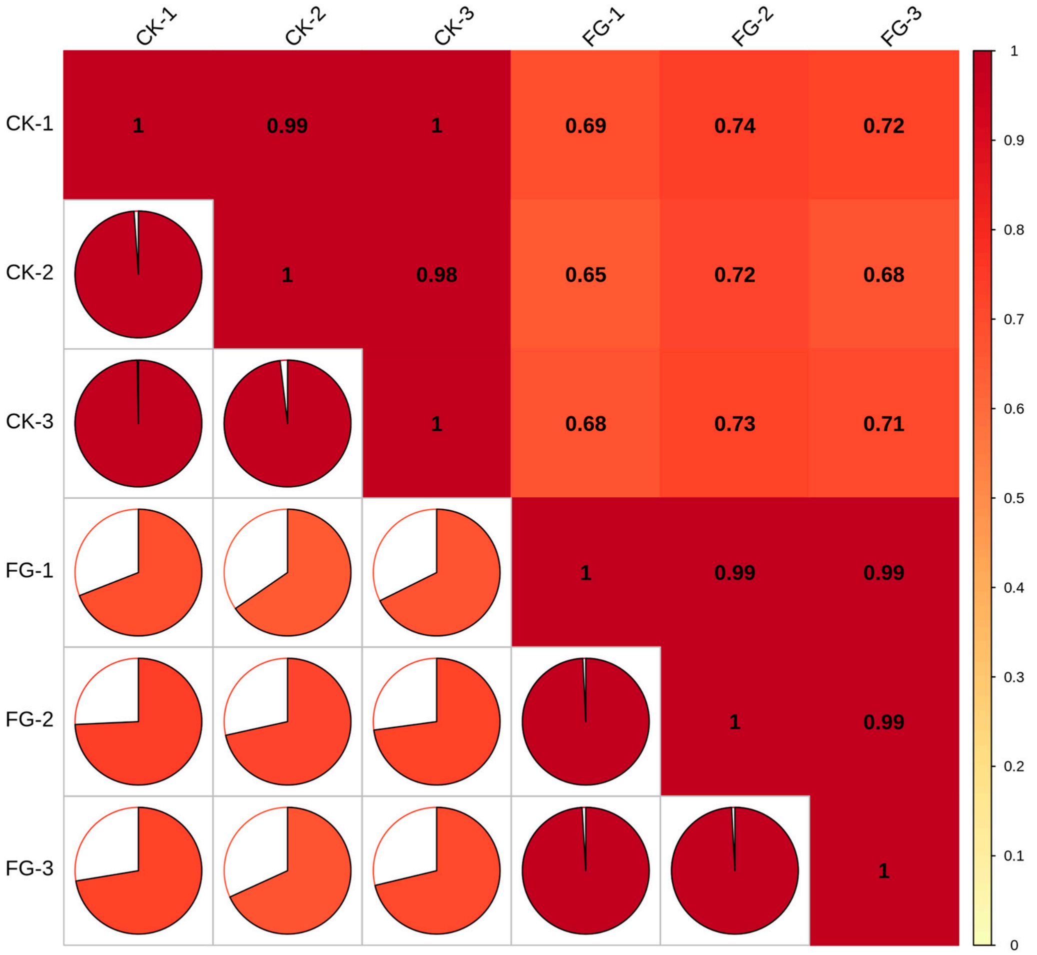

Figure S2. Sample correlation graph of transcriptome sequencing. Pearson's Correlation Coefficient (r) was used as an evaluation index of biological repeat correlation. The closer the absolute value of r is to 1, the stronger the correlation between the two duplicate samples.

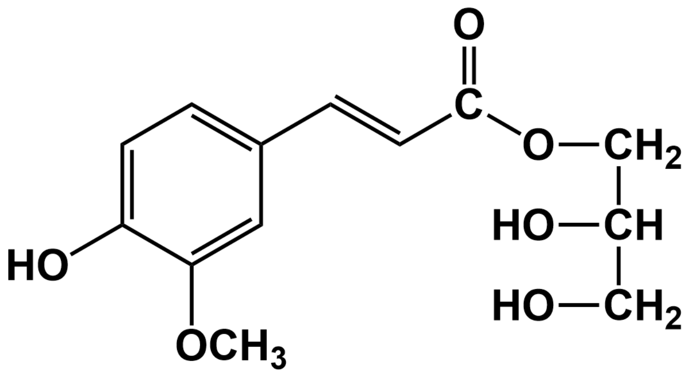

Figure S3. Chemical structural formula of FG.

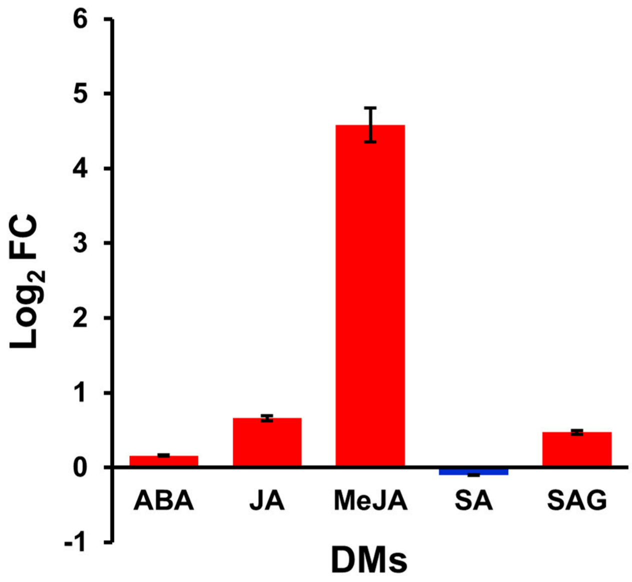

Figure S4. Effects of FG on defense hormones. Metabolic levels of defense hormones such as abscisic acid (ABA), jasmonic acid (JA), methyl jasmonate (MeJA), salicylic acid (SA) and salicylic acid-2-O-β-glucoside (SAG) under FG treatment.
