# Supplementary material for: Feruloyl Glyceride Mitigates Tomato Postharvest Rot by Inhibiting Penicillium expansum Spore Germination and Enhancing Suberin Accumulation
Source: Foods. 2024 Apr 10;13(8):1147. doi: 10.3390/foods13081147 (PMC11049243; doi:10.3390/foods13081147)
Supplement: Supplementary file 1 [file foods-13-01147-s001.zip › Table S1. The sequence of qPCR primers.pdf]

Table S1. The sequence of qPCR primers

Gene	F / R	Sequence（5'-3'）
SlActin	F	CGAGCAGTGTTTCCCAGTATT
	R	AGCCTGGATAGCAACATACATAG
18SrRNA（18Sr）	F	ATTGGAGGGCAAGTCTGGTG
	R	CCAGTGAAGGCCATGGGATT
SlUBI	F	AGGTTGATGACACTGGAAAGGTT
	R	AATCGCCTCCAGCCTTGTTGTA
SlPAL1	F	GGTGAGAAACTTGACGCTCAT
	R	GCTGCAACTGGAAAAATCCACC
SlLACS8	F	CTGGGAAGGGGCTACAACTG
	R	ACTCCCACTGATACTCCCCC
SlFAR4	F	ACTCCGCAGCAAATACCACA
	R	GCGGTCGAATAATGGATGAGG
SlKCS11	F	CCAACACCCTCTCTGTCCTC
	R	TGGATGCTCTCCGTGCTAAC
SlCYP86A1	F	AGCCACTGAAGCCACTATGC
	R	CCTTACGCGAATCCAAAGCC
SlCYP86B1	F	TTCGGAACATGACGGCAGAT
	R	CTGGATCAACCCCAAAGGCA
SlGPAT5	F	CGGCTAGAGGATGGAAAGGG
	R	GACTCGATTTCCCCGCGTTA
SlPPO	F	GTTGGTGGTCTTCCCTTCGT
	R	ATGACGACGGAAGCTGGAAG
SlLOX5	F	ATCCGAACCGATGGTGAAGG
	R	AGGATTTACTCCGGCAAGCA
